# Public health genomics and personalized prevention: lessons from the COGS project

**DOI:** 10.1111/joim.12094

**Published:** 2013-10-16

**Authors:** N Pashayan, A Hall, S Chowdhury, T Dent, P D P Pharoah, H Burton

**Affiliations:** 1 Department of Applied Health Research, University College LondonLondon, UK; 2 PHG FoundationCambridge, UK; 3 Departments of Oncology and of Public Health and Primary Care, University of CambridgeCambridge, UK

**Keywords:** ethical, legal and social issues, personalized prevention, polygenic risk stratification, public health genomics, screening

## Abstract

Using the principles of public health genomics, we examined the opportunities and challenges of implementing personalized prevention programmes for cancer at the population level. Our model-based estimates indicate that polygenic risk stratification can potentially improve the effectiveness and cost-effectiveness of screening programmes. However, compared with ‘one-size-fits-all’ screening programmes, personalized screening adds further layers of complexity to the organization of screening services and raises ethical, legal and social challenges. Before polygenic inheritance is translated into population screening strategy, evidence from empirical research and engagement with and education of the public and the health professionals are needed.

## Introduction

The success of the Collaborative Oncological Gene-environment Study (COGS) 1 in identifying multiple new genetic variants associated with the risk of breast, prostate and ovarian cancers promises a new era in personalized prevention. By stratifying the population into several groups according to genetic risk alone or combined with traditional disease risk factors (such as age and family history), standard public health interventions could be applied differentially to each population stratum with potentially more efficient outcome 2,3. However, the challenge remains how to translate genomic knowledge into health improvement programmes and disease prevention interventions at the population level. Responding to such a challenge represents the core work of public health genomics.

Public health genomics is a relatively new discipline that emerged in 1997 when small units were established independently on both sides of the Atlantic – the Office of Genetics and Disease Prevention at the Centers for Disease Control and Prevention in Atlanta, USA, and the Public Health Genetics Unit in Cambridge, UK. Subsequently, following a multidisciplinary expert meeting supported by the Rockefeller Foundation at Bellagio, Italy, in 2005, public health genomics was formally defined as ‘the responsible and effective translation of genome-based knowledge and technologies for the benefit of population health’, and the broad content and methodology of this discipline was specified 4,5. It was stated at the Bellagio workshop that improving population health would require an integrated and multidisciplinary approach, taking account of emerging genome-based science and technologies and integrating such knowledge with the humanities, social sciences and population sciences, particularly epidemiology. An important part of the definition of public health genomics was that interventions arising from genomic research should not be introduced into clinical or public health practice prematurely without the support of a solid evidence base. The element of ‘responsibility’ in the definition demonstrated the importance of the societal context in which new technologies will be applied and the collateral effects on individuals and populations. Finally, the definition encompassed the view that implementation of new genetic applications would require an active process, involving multidisciplinary analysis, public dialogue and involvement, engagement with a wide range of stakeholders, informing public policy, developing new programmes and services, strategic planning and evaluating health services, and training and educating health professionals, policy makers and the public.

In this review, we illustrate the potential utility of polygenic risk stratification in prevention programmes at a population level using the case of population-based screening for breast and prostate cancers 6. Using the principles of public health genomics, we examine the challenges of implementing personalized screening programmes. These analyses were carried out as part of the Work Package 7 of the COGS project funded by the Seventh Framework Programme of the European Commission.

## Polygenic risk stratification

The risk alleles identified by genome-wide association studies individually confer a modest increase in risk of disease (usually per-allele relative risk of less than 1.5). Consequently, the predictive utility of a genetic test based on a single risk allele is poor. Even using a combination of multiple alleles, the clinical utility of a polygenic test in predicting future disease for the individual will be limited 7. This is because most individuals are at only slightly increased or decreased risk, and the difference is modest even for the small number at the extremes of the distribution. However, discrimination (i.e. determining which individuals will or will not experience disease) is not the only measure of clinical utility of a risk prediction model. In disease prevention, the aim is to stratify risk rather than to discriminate events 8.

According to the multiplicative model, the polygenic risk in the population at birth follows the normal distribution when relative risk is plotted on a logarithmic scale. The normal distribution is defined by its mean and variance or spread 9. The variance of the distribution of polygenic risk is calculated from the risk allele frequencies and per-allele relative risk 2,10. The distribution of relative risk amongst cases is also log-normal with the same variance as the population, but with a larger mean; cases are on average at a higher prior risk of developing cancer than the population 9. Given the variance and mean of the distribution of the log-normal relative risk, the percentile rank associated with a given polygenic relative risk (or age-conditional absolute risk) threshold in the population or in cases can be calculated 6.

## Utility of polygenic risk stratification for personalized screening

We modelled the number of individuals eligible for screening and the number of cases potentially detectable by screening in a population undergoing screening based on age alone, as compared to a population undergoing personalized screening based on the 10-year absolute risk of being diagnosed with breast or prostate cancer. We calculated the conditional absolute risk taking into account age and polygenic risk profile. We set the risk threshold equivalent to the threshold for eligibility in the age-based screening programme 6.

In the case of breast cancer, we estimated the polygenic risk based on 67 common genetic susceptibility variants [11, which confer a polygenic variance of 0.28 and explain approximately 14% of the genetic component of breast cancer risk. Compared with the UK National Health Service Breast Screening Programme, which offers screening to women aged 47 to 73 years (10-year absolute risk of being diagnosed with breast cancer of 2.5% or greater), personalized screening of women aged 35 to 79 years at the same risk threshold would result in 24% fewer women being eligible for screening whilst potentially detecting 3% fewer cases.

In the UK, there is no national screening programme for prostate cancer, but a similar approach could be applied if there were. We estimated the polygenic risk of prostate cancer based on 72 common susceptibility variants 12, which confer a polygenic variance of 0.44 and explain approximately 30% of the genetic component of prostate cancer risk. Compared with a hypothetical screening strategy based on age alone in which men are eligible for screening from age 55 to 79 years (10-year absolute risk of being diagnosed with prostate cancer of 2% or greater), personalized screening of men aged 45 to 79 years at the same risk threshold would result in 19% fewer men being eligible for screening at a cost of 4% fewer potentially screen-detectable cases.

The efficiency of personalized screening will improve as more susceptibility variants are known. Based on the currently known susceptibility variants for breast cancer, 43% of 35- to 79-year-old women would be eligible for screening with 71% of cases being potentially screen detectable. In a hypothetical best-case-scenario analysis, assuming all possible susceptibility variants for breast cancer were known (predicted polygenic variance of 1.44 9,13), 28% would be eligible for screening whilst potentially detecting 76% of cases. For prostate cancer, based on the currently known susceptibility variants, 51% of 45- to 79-year-old men would be eligible for screening whilst potentially detecting 92% of cases. In the best-case-scenario (predicted polygenic variance of 1.58), 34% would be eligible whilst potentially detecting 89% of cases (Table 1).

**Table 1:** The likely percentage of the population eligible for screening and of the cases potentially detectable by screening, considering age-based and personalized screening for breast and prostate cancers with increasing numbers of known susceptibility variants

Screening strategy	Population eligible for screening (%)	Cases potentially detectable by screening (%)
	Age 35–79 years; *n *= 13 126 890	Age 35–79 years; *n *= 30 936
Breast cancer		
Age-based screening (47–73 years)	57	72
Personalized screening (age 35–79 years and 10-year absolute risk ≥2.5%)		
Currently known variants (variance* *= 0.28)	43	71
Variants explaining 50% of familial risk (variance* *= 0.72)	35	72
Variants explaining 100% of familial risk (variance* *= 1.44)	28	76
	Age 45–79 years; *n *= 8 655 126^a^	Age 45–79 years; *n *= 22 836^a^
Prostate cancer		
Age-based screening (55–79 years)	63	96
Personalized screening (age 45–79 years and 10-year absolute risk ≥2.0%)		
Currently known variants (variance* *= 0.44)	51	92
Variants explaining 50% of familial risk (variance* *= 0.79)	45	91
Variants explaining 100% of familial risk (variance* *= 1.58)	34	89

a Estimates are based on the population and cancer registrations in 2002–2006 in England.

**Figure 1:**
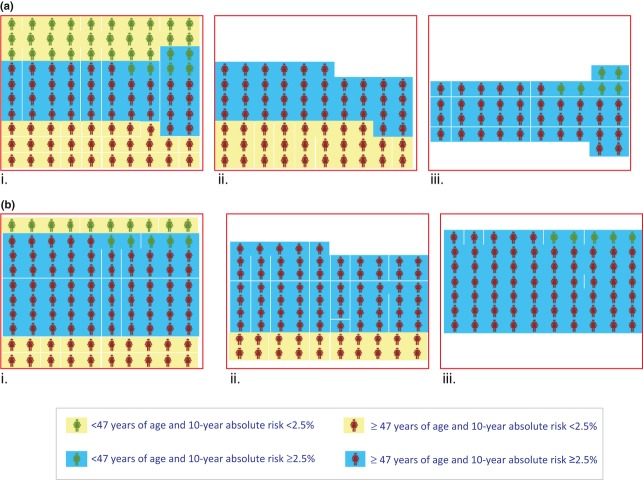
Reclassification of women into different risk groups. a, Eligibility for screening: a population of 100 women, 35–79 years of age, by age group and risk threshold (i), eligible for screening based on age alone (ii) or on age and polygenic risk (iii). b, Potentially screen-detectable cases: a population of 100 women, 35–79 years of age, with breast cancer by age group and risk threshold (i), detectable by screening based on age alone (ii) or on age and polygenic risk (iii).

Figure 1 shows how stratification based on the absolute risk that is dependent on age and polygenic risk profile reclassifies individuals into different risk groups, whereby some cancers that would have been detected under age-based screening will not be detected by personalized screening and vice versa. Such reclassification may improve the benefits and reduce some of the harms associated with screening. A personalized screening programme would enable the detection of cancer in younger individuals who are at high risk. Prostate and breast cancers detected in younger individuals tend to behave more aggressively 14,15. So detecting these cancers amongst younger individuals at high risk may improve their prognosis. If polygenic low risk is associated with indolent and possibly overdiagnosed cancer, then not detecting cancer amongst individuals at low risk may potentially reduce the harms associated with overdiagnosis and overtreatment.

Under an age-based screening strategy, all individuals within the age range will be eligible for screening, whereas individuals will be invited for screening at different ages based on their absolute risk level under a personalized screening strategy. For example, in the case of screening for prostate cancer, instead of inviting all 55- to 79-year-old men, 2%, 17% and 96% of men aged 47, 50 and 65 years, respectively, would be invited for screening. Thus, different individuals will undergo different numbers of screening episodes during their lifetime. A reduction in the number of individuals offered screening may reduce the number of false-positive screens, with a reduction in the harms associated with false-positive results and the benefit of saving further resources on diagnostic tests.

However, to estimate the true benefits of personalized screening, we need to understand whether and how tumour subtypes, screening test sensitivity, the natural history of cancer and the probability of overdiagnosis vary by polygenic risk profile. Further evidence from empirical data is needed.

## Challenges of implementing personalized screening

In addition to considering the utility of polygenic risk stratification for personalized screening, there are many other issues to consider before a risk-tailored programme could be put into practice. These are interdependent and include cost-effectiveness, public and professional acceptability, and organizational and a wide range of ethical, legal and social issues.

### Evaluation of cost-effectiveness

A screening programme becomes viable if it does more good than harm at a reasonable cost 16. A personalized screening strategy involves the additional cost of polygenic risk profiling set against the potential savings from repeat screening and diagnostic work-up of false-positive results. Our preliminary cost–utility analysis comparing age-based screening to a personalized screening programme for prostate cancer indicates that personalized screening would cost less and would increase the number of quality-adjusted life years gained.

### Exploring organizational and ethical, legal and social issues

We convened international multidisciplinary stakeholder workshops, followed by ongoing detailed research and policy analysis of the key issues identified 17–19. The workshop participants included clinicians, geneticists, public health specialists, epidemiologists, ethicists, lawyers, social scientists, screening programme managers, a journalist and public representatives. In addition to panel discussions and small working groups, we used hexagon modelling, a systematic technique developed by IDON 20, to brainstorm, capture and determine the priority of the issues raised. The key issues are summarized below.

An overriding concern was whether eligibility for screening based on a risk score that includes genetic profiling alongside environmental and lifestyle risk factors would be acceptable to the public, health professionals and policy makers. Particularly where there is already an established screening programme, such as that for breast cancer in the UK, not or less frequently offering screening to lower-risk groups may be perceived as service rationing and unfair discrimination based on a genetic profile that is unalterable and therefore not under control of the individual. The DNA testing involved in risk profiling may itself cause concerns amongst the public about who might access these data and whether they could be used for purposes other than for health care or public health, such as for medical research or forensic investigation, or to inform decisions about insurance or employment.

The greatly increased complexity of a polygenic risk-based screening programme could result in a further set of issues. These are evident in both the design and delivery of the programme. A major organizational challenge will be to incorporate the advances of the rapidly evolving field of genomics and the changes in individuals’ environmental and lifestyle risk factors, including their family history of disease, into a dynamic risk estimation tool 21. In terms of the environmental and lifestyle risk factors, it is critical to determine how, when, with what frequency and by whom this information will be obtained and updated. For the genetic data, it will be necessary to decide the age of sampling and testing, details of the variants to be included, whether rare highly penetrant mutations such as *BRCA1/2* will be included and whether to use a bespoke set of variants related to the particular condition or to use whole-genome sequencing for a panel of common chronic diseases. In the latter case, the relevant information regarding prevention programmes could be extracted as and when required 22.

The programme design also needs to set out the different patterns of screening for which people in differing risk strata will be eligible. Depending on further research evidence, lower-risk groups may be offered screening less frequently and higher-risk groups may be offered screening with more sensitive (and possibly more expensive) tests, such as with magnetic resonance imaging instead of mammography for breast cancer screening 23. To adapt services already in place or to develop new ones, substantial organizational effort will be required supported by a range of evaluative research to ensure that the level of extra value merits the additional complexity and cost.

Finally, the delivery of the programme and, in particular, the interaction of those at the frontline of provision with participants must be considered 24. Individuals need to be able to make an informed decision about whether to accept an offer to participate. To support this and to protect the public, it is important to establish policies, for example, regarding: (i) the amount and type of information to be provided to the public, (ii) how the different levels of risk will be communicated, (iii) the potential benefits and possible harms of screening interventions for different risk strata and (iv) regulations on data safeguarding. Health professionals involved in service delivery will need training to better understand how the risk scores are determined and the evidence on which different management options are based, to communicate these effectively and to be able to provide support to patients at various stages of the programme and with different outcomes.

## Conclusion

Polygenic risk stratification would potentially improve the effectiveness and cost-effectiveness of screening programmes. However, in terms of set-up and delivery, a risk-tailored screening programme is much more complex than a programme with eligibility based on age alone. Any decision to initiate a personalized screening programme should first address the organizational and ethical, legal and social issues and commit to public engagement and education and to work with the health professionals delivering the programme.

From a wider perspective, the COGS project illustrates the enterprise of public health genomics. We have shown how the practice of public health genomics builds on a detailed understanding of the underlying science and technology and incorporates elements of population sciences such as epidemiology, health economics and consideration of ethical, legal and social issues as well as policy development and change management.

We have illustrated the complexity of knowledge that must be integrated and the detailed planning, consultation and development work that would be required to underpin such a new programme. As the definition of public health genomics suggests, the ‘responsible translation of genomic technology for the improvement of population health’, in its widest sense, will demand no less.

## Conflict of interest statement

The authors have no conflict of interests to declare.
